# The Tongue

**Published:** 1881-08

**Authors:** J. Milner Fothergill


### The Tongue.

By J. Milner Fothergill, M. D.

Much may be learned from accurate observation of the tongue; how much, a few old practitioners almost alone can tell. In the treatment phthisis, inspection, minute and scrutinizing, of the tongue is far more important than the wielding of the stethoscope, however skillfully. The one tells much of the amount and nature of the disease, the latter gives information, often priceless, as to the precise line of treatment to be adopted; for the tongue is the index of the state of the intestinal canal, and if the *primæ viæ* are disordered, they must be put right before any other therapeutic measure can be safely adopted.

Tell the patient to put out his tongue fully, so that the circumvellate papillæ can be clearly seen; it is no use to study the tip. If the patient is an infant, Sir William Jenner's plan of placing a drop of fluid, especially if viscid as syrup, upon the chin, is well worth following. A tickling sensation is produced, and the little patient tries to remove the cause of irritation with its tongue. The condition of the tongue can thus be studied without much disturbance to the child. The manner of protrusion is instructive. In the typhoid condition of fevers, and in some cerebral affections, the request to put out the tongue has to be repeated, and loudly, before the patient does as is requested; and similar reiteration is requisite to induce its withdrawal. "It is a curious fact that patients will frequently protrude the tongue when they cannot be made to do aught else, owing to the state of their mental faculties" (Flint). Then tremulousness of the tongue indicates alcoholism; and less frequently lead or mercurial poisoning. Tremulousness of the tongue may denote muscular weakness. When seen in the early stages of typhus, or typhoid fever, it indicates a grave condition of bad prognostic omen. In advanced stages the tongue is protruded slowly and with difficulty, indicating impaired power over the muscles. In hemiplegia the tongue, when protruded, turns its apex to the paralyzed side, from diminution of power in the genio-hyoglossus muscles of the affected side. In bulbar, otherwise glosso-labial paralysis, the capacity to protrude the tongue is impaired or lost. In facial paralysis, without hemiplegia, this loss of power to portrude the tongue tells that the mischief is central, and within the skull; and not peripheral, or Bell's palsy.

Dryness of the tongue is found in pyrexia, whether the fever be specific or symptomatic. It is also dry in diabetes, and other conditions of polyuria, and in some of the functional disorders of digestion. It becomes dry and hard, as well as brown, from the accumulation of dead epithelium cells upon it in the typhoid condition and in uræmia. When the mouth is kept open, it becomes dried, as is seen in some forms of dyspnœa. Then it is edentated; and marked by the teeth in conditions of debility, from menorrhagia, chronic diarrhœa, or acute prostration, however induced. Then as to the state of the tongue known as "coated" or "furred." This is constant with some individuals who are well and strong; and a furred tongue, especially in the morning, is common with heavy smokers. But usually a furred tongue denotes disturbance of the digestive organs, or the oncome of acute disease, especially the specific fevers. When found with shivering fits, this condition of the tongue tells of coming trouble. When the coating has a distinctly yellow or brownish hue, there is usually a bad taste in the mouth in the morning when awakening; the taste and the color are both due to tauro-cholic acid. This is denied by some authorities, who say there is no connection betwixt the state of the tongue and the condition of the liver; but the great bulk of medical experience is dead against them. The fur on the tongue consists mainly of dead epithelium cells, mucus, particles of food, and dust inhaled by the breath. As the rude index of the condition of the gastro-intestinal canal, the state of the tongue furnishes valuable information. Where the coat is thick, it is evident that absorption of food from the intestines must be very imperfect through the layer of epithelial cells; and our efforts are directed to remove this obstructive layer. Consequently we inspect the tongue in acute disease, and in convalescence, in order to ascertain, with such an approach to accuracy as the tongue can tell us, whether the state of the intestinal canal is such as will permit of the assimilation of the injesta. When the tongue cleans, then we know assimilation is going on satisfactorily. When the tongue remains coated, we aid the natural efforts to remove the fur by a mercurial laxative. Repeated free purgation without a mercurial often leaves the tongue as thickly coated as before; and a few grains of calomel produce a clean tongue in a few hours. At other times a dose of calomel may get the credit of cleaning the tongue, when it is due to a natural process. I remember well the case of a boy who had been threatened with enteritis. He was progressing nicely, but the tongue did not clean; I spoke of giving him a powder, but counter-ordered it. Next day he had two free semi-fluid motions, and the tongue was quite clean. Had the powder, which would have consisted of three grains of calomel, been given, it would unquestionably have got the credit of producing the change. This was years ago, in the early days of general practice, but the lesson has never been forgotten. It is always well to see the tongue clean; and in private practice more attention is, and has to be, paid to the state of the tongue than is given to it in hospital practice usually. In acute disease the mucous membrane commonly is unequal to shedding its dead epithelium, and when the shedding occurs, it is a good omen of returning vigor. In protracted illness the fur may be shed and reproduced again several times. After acute disease, and especially fevers, the fur may disappear bit by bit, commencing at the tip, and creeping along the edges; leaving a thick coat up the mesial line and upon the base, which in time also disappears. Such clearing up of the tongue is of the best prognostic omen, and tells of uninterrupted convalescence. In scarlet fever the tongue assumes a "strawberry" appearance; sometimes the red papillæ stand out on a red surface like a ripe, red strawberry, at other times the red papillæ stand out upon a coat of fur, like the seeds on an unripe white strawberry. A furred tongue is manifested in many cases of dyspepsia, especially when many "by-products" of digestion are formed in the digestive act. Both in indigestion and artificial digestion there are by-products formed as well as peptones, and these "by-products" are offensive and objectionable. In some cases of acid heartburn the chief offending agent is butyric acid. In almost every case of indigestion with a furred tongue constipation is present, and must be considered in the therapeutic plan. Here nothing but a continuous course of laxatives, and occasionally acute purgation at intervals, will be of any service; and the treatment must be continued, no matter how long, until the system rights itself. In some cases the patience of doctor and patient becomes severely tried, but perseverance brings with it at last its reward. All mechanical means of cleaning the tongue, as scraping it, or rubbing it with lemon-juice or vinegar, are well enough for the local sense of cleanliness and comfort, especially in pyretic states; but they are utter rubbish and nonsense as to cure, which depends upon other measures altogether.

Then the tongue may be furred along one side only, or may be raw and irritated, or even ulcerated, by a decayed tooth with a jagged edge. At other times the epithelium of the tongue is stained, as by drinking elder wine, sucking a piece of licorice, or chewing tobacco; or it may be discolored by some preparation of iron. These modifications of its appearance are more distracting and puzzling when the tongue is coated with fur pretty thickly.

The "raw" or "bare" tongue. This condition of the tongue has not, in my experience, received from medical writers a tithe of the attention it deserves to have paid to it. Here the superficial structures of the tongue are denuded, more or less completely, of the natural epithelium. In convalescence from acute conditions, where the tongue has been coated, sometimes the tongue is abnormally red and imperfectly covered with epithelium, and here a coat is apt to form again (Flint). Both in acute or chronic conditions, the absence of the normal epithelial covering, whether slight or considerable, should receive the keenest attention of the practitioner. As long as the tongue is "raw" or "bare," the line of treatment to be followed is that of bland food, with sedatives to the gastro-intestinal tract, as bismuth, with alkalies, or opium, or both. So long as this condition remains, tonics are useless, and are not digested. At the risk of being charged with dogmatism, I venture to insist upon this. Perhaps it is in phthisis, of all diseases, where this rawness of the tongue excites one's apprehensions; at least, it is of all semeia the one I personally dislike most. It is not usually complete over the whole tongue, but lies as a large patch in the middle of the tongue, the irregular edge usually extending further on one side of the mesial line than on the other. We have every reason for supposing that this condition of tongue is significant of the state of the unseen portion of the gastro-intestinal canal; and the absence of epithelium interferes with assimilation. This it is which excites one's apprehension in all wasting diseases.

"After a meal the epithelium cells of the villus are found crowded with fat. Since the stiration of the hyaline border of the cells is not due to pores, as was once thought, the particles must have entered into the cells very much as foreign particles enter the body of an amœba. The epithelium may, in fact, be said to eat the fat" (Michael Foster). If, then, the epithelial layer be defective from absence of epithelium, or from the epithelial cells being imperfectly developed, and therefore functionally defective, fat cannot be properly absorbed; and that absorption of fat is of all things what we especially desire in wasting disease. Not only is the epithelial layer important in the absorption of nutritive material from the food in the intestines, but it is essential to secretion. "The food, in passing along the alimentary canal, is subjected to the action of certain juices which are the products of the secretory activity of the epithelium cells of the alimentary mucous membrane itself, or of the glands which belong to it. These juices (*viz.*, saliva, gastric juice, bile, pancreatic juice, succus entericus, and the secretion of the large intestine,) poured upon and mingling with the food, produce in it such changes, that from being largely insoluble it becomes largely soluble in an alkaline fluid such as blood, or otherwise modify it in such a way that the larger portion of what is eaten passes into the blood, either directly by means of the capillaries of the alimentary canal, or indirectly by means of the lacteal system, while the smaller part is discharged as excrement" (Foster). Now, if "the epithelium cells of the alimentary canal play this important part in the digestive act, it is quite obvious and abundantly clear that deficiency in number or perfection of these epithelium cells must exercise a deep and profound influence upon digestion, absorption, and nutrition." That all the practitioner's energies should be bent towards the restoration of the epithelial layer to normal perfection, or the best approach thereto, is intelligible enough; and the attention paid to the *primæ viæ* by our predecessors was amply justified by its importance. It is, too, comparatively easy to get rid of the layer of dead epithelium cells of the coated tongue; but it often taxes all our resources to restore the epithelial coat to its integrity where the tongue is "raw" or "bare." Yes, and sadly, too often our best efforts are futile and unproductive of good result! When the "bare" tongue is the index of a deficient epithelial layer in the alimentary canal—and no other index do we possess—the first duty of the practitioner is to do his utmost to restore it to the normal condition. How this is to be achieved will be considered hereafter. When, then, under appropriate treatment the tongue assumes its normal appearance, and the epithelium once more grows freely upon it, then we know the digestive powers are returning, and that we may venture on tonics, and more food of a less restricted character. A shrewd practitioner, young or old, will always study the condition of the epithelial layer of the tongue carefully, sagaciously, with a full knowledge of what is revealed by the condition of that part only of the alimentary canal which is open to our vision. So long as the condition of "raw" or "bare" tongue continues, so long must our therapeutic measures be directed to the restoration of the epithelial layer of the alimentary canal to its integrity—or the nearest attainable approach thereto. Then the tongue may present a "beefsteak" appearance when it is denuded of epithelium, as it is apt to do when the brown fur of the typhoid, or uræmic condition has been shed. The system is equal to shedding the dead epithelium, but it is not quite equal to the production of a new layer of perfect epithelium.

The epithelium layer of the tongue is often suggestive of other conditions than those of the alimentary canal. There is a peculiar silvery sheen of the epithelial covering of the tongue in many cases of menorrhagia; especially when the tongue looks swollen and shows the indentation of the teeth. I have nothing to say as to the "how" of this association; but it is certainly sufficiently common to give this condition of the tongue a distinct diagnostic value.

In relapsing fever there is often a small triangle on the tip of the tongue, much cleaner, or "rawer," than the rest of it. Each side of this equilateral triangle is about half an inch in length. I have seen it both here and in Germany.

Then the surface of the tongue may be altered. The mucous membrane may be ulcerated, as in stomatitis. Glossitis it is not my province to describe. Or it may be fissured. Deep rugour fissures are very suggestive of syphilis. So is a large bare patch with or without fissures, without acute disturbance of the health; while patches of syphilitic psoriasis where the affected epithelial scales are shed and a bare patch is left, are not at all uncommon. The tongue may be the seat of a chancre, which must be discriminated from cancer; this is done by the history, the age, and the conditions of the glands of the neck. When inspecting the tongue, other evidences of syphilis may be furnished by the state of the pharynx, or soft palate. Then the tongue may be indented by the teeth. "Indentations on the margins may be produced by the pressure of the teeth. These occur if the organ be swollen; otherwise they simply show that it has remained in contact with the teeth for a considerable time. In health, during wakeful hours, it is frequently moved, not remaining, except momentarily, in the same place. The indentations due to diminished movements denote mental hebetude. The tongue occasionally presents fissures or cracks in the course of fevers, and these sometimes continue into convalescence. Cicatrices are observed in persons subject to epilepsy, as the result of wounds inflicted by the teeth during the paroxysms. These may be useful in determining that paroxysms which a patient has experienced were epileptic in character. Coldness of the tongue belongs to the moribund condition, without reference to the disease, and it is a striking symptom in the algid stages of epidemic cholera" (Flint).

A tongue fissured not deeply, but with many little fissures over its surface, I have very commonly noted in persons who drink their tea very hot; but it is not invariably, though commonly, so associated.—*Leonard's Illus. Med. Journal.*

